# Development of Nano Soy Milk through Sensory Attributes and Consumer Acceptability

**DOI:** 10.3390/foods10123014

**Published:** 2021-12-05

**Authors:** Seyoung Ju, Sooji Song, Jeongnam Lee, Sungwon Hwang, Yoonmi Lee, Yongseok Kwon, Yuyoung Lee

**Affiliations:** 1 Department of Integrated Biosiences, College of Biomedical and Health Science, Konkuk University, Chungju-si 27478, Korea; syoungju86@kku.ac.kr (S.J.); ssj4037@naver.com (S.S.); yoonmilee@kku.ac.kr (Y.L.); 2 Sahmyook Food R&D Center, Seongjin-ro, Cheonan-si 31033, Korea; Ginoo76@hanmail.net; 3 Department of System Semiconductor Engineering, Sangmyung University, Sangmyeongdae-gil, Cheonan-si 31066, Korea; sungwon@smu.ac.kr; 4 National Institute of Agricultural Sciences, 166 Nongsaengmyeong-ro, Wanju 55365, Korea; selenium2012@korea.kr; 5 Department of Central Area, National Institute of Crop Science, Rural Development Administration, Suwon-si 16429, Korea

**Keywords:** sensory attributes, consumer acceptability, laboratory-developed soy milks, partial least square regression (PLSR) analysis

## Abstract

Nanotechnology is currently applied in food processing and packaging in the food industry. Nano encapsulation techniques could improve sensory perception and nutrient absorption. The purpose of this study was to identify the sensory characteristics and consumer acceptability of three types of commercial and two types of laboratory-developed soy milk. A total of 20 sensory attributes of the five different soy milk samples, including appearance, smell (odor), taste, flavor, and mouthfeel (texture), were developed. The soy milk samples were evaluated by 100 consumers based on their overall acceptance, appearance, color, smell (odor), taste, flavor, mouthfeel (texture), goso flavor (nuttiness), sweetness, repeated use, and recommendation. One-way analysis of variance (ANOVA), principal component analysis (PCA), and partial least square regression (PLSR) were used to perform the statistical analyses. The SM_D sample generally showed the highest scores for overall liking, flavor, taste, mouthfeel, sweetness, repeated consumption, and recommendation among all the consumer samples tested. Consumers preferred sweet, goso (nuttiness), roasted soybean, and cooked soybean (nuttiness) attributes but not grayness, raw soybean flavor, or mouthfeel. Sweetness was closely related to goso (nuttiness) odor and roasted soybean odor and flavor based on partial least square regression (PLSR) analysis. Determination of the sensory attributes and consumer acceptance of soymilk provides insight into consumer needs and desires along with basic data to facilitate the expansion of the consumer market.

## 1. Introduction

Soy milk is a plant beverage with high nutritional value due to the abundance of proteins, fatty acids, and health ingredients, such as isoflavones, oligosaccharides, anthocyanins, and dietary fiber. Many studies have reported the antioxidant, anticancer, antidiabetic, and hypocholesterolemic activities of soy milk in addition to its therapeutic effects against osteoporosis, kidney disease, and high blood pressure [1,2,3,4,5,6]. Therefore, there has been an increased interest in nutritional aspects and demand for soy products in the beverage industry. In America, sales of soy milk increased dramatically from $500 million in 2001 to $1 billion in 2008 [7]. Despite the rapid increase in the sales of soy milk, it is purchased by 13% or fewer households in the USA [8]. Soy-based beverages accounted for 7.7% of the market share in Korea compared with 22.8% attributed to fruits and vegetables juice, with coffee drinks accounting for 17.5%, soft drinks for 16.4%, functional beverages constituting 11.0%, and mineral water amounting to 10.9% of the total beverage market in 2012 [9]. Nevertheless, some consumers do not appreciate soymilk because of the raw-beany flavor and the unique taste [10].

Soy bean flavor and nutrients are affected by a number of factors, such as soy bean cultivars, geographic differences, and manufacturing techniques, as well as the soy bean components including proteins, fatty acids, oligosaccharides, and other contents [10,11,12]. The technology for the improvement of soy bean flavor ensures the retention of key nutrients, which contribute to the popularity of the beverage worldwide. Studies have investigated the analytical and sensory attributes of soy milk, and the consumer test results [3,6,12,13,14,15,16,17,18,19,20].

However, the relationship between sensory analysis and the effects of processing methods have rarely been reported. Nanotechnology is a rapidly growing area for the manufacture of nanoscale materials. Nanotechnology can be applied to food packaging, food processing, and functional foods to ensure food safety and quality [21]. Nanotechnology in the food industry could currently be applied to protect foods against bacterial deterioration and extend the shelf life of foods [22], increase the bioavailability of bioactive compounds and nutrient absorption [23,24], protect against oxidant ingredients [25,26], and also increase sensory perception [27]. Some studies have shown that nanomaterials by delivery systems improved the bioavailability of bioactive compounds, such as calcium [24], vitamins [28], and iron [29].

Although research on food nanotechnology has yet to be fully clarified, it could affect the sensory perception for improving flavor release and flavor retention by nanoencapsulation techniques [30]. Nanotechnology can be used to improve food characteristics, such as particles and flavor, and foods with nanomaterials show higher production efficiency, better taste, and nutrient absorption [22]. Soy milk products generally have some problems, such as raw-beany flavor and residual aftertaste, even if they have high nutritional values. Therefore, we would like to develop nano soy milk, which could have low beany flavor and residual taste with nano particles using nanotechnology.

This study investigated the descriptive sensory characteristics and consumer acceptance of three commercially available and two laboratory-developed types of soy milk using nanotechnology and analyzed the relationship between descriptive sensory characteristics and consumer acceptability of five types of soy milk. The differences in the sensory attributes and consumer acceptability of soymilk can be used to analyze the consumer needs and desires. Therefore, these results provide basic data for the market expansion of soy milk.

## 2. Materials and Methods

### 2.1. Materials

Five types of soy milk were investigated in this study. The products were named SM_W (Soy Milk_Woori), SM_Y (Soy Milk_Y brand), SM_D (Soy Milk_D brand), SM_N (Soy Milk_Nano), and SM_J (Soy Milk_J brand). Three (SM_Y, SM_D, and SM_J) stable and commercially available products were sugar-free and contained no additives. The other two types of soy milk were synthesized in the laboratory. The soy milk types developed in the laboratory (SM_W and SM_N) were derived from the new soy bean crop (Daechan, Chungja 3, Saedanbaek) grown in 2015 and 2016 by the National Institute of Crop Science of Rural Development Administration in Korea. The Chungja 3 soybean includes additional isoflavone and anthocyanin content, and the Saedanbaek soybean contains a higher protein content compared with the general soybeans. One of the synthetic soy milks (SM_N) was composed of nanoparticles, which increased the digestion and absorption compared with the general soy milk. Table 1 lists the sample names, brands, and ingredients.

### 2.2. Soy Milk Production and Sample Preparation

**Figure 1:**
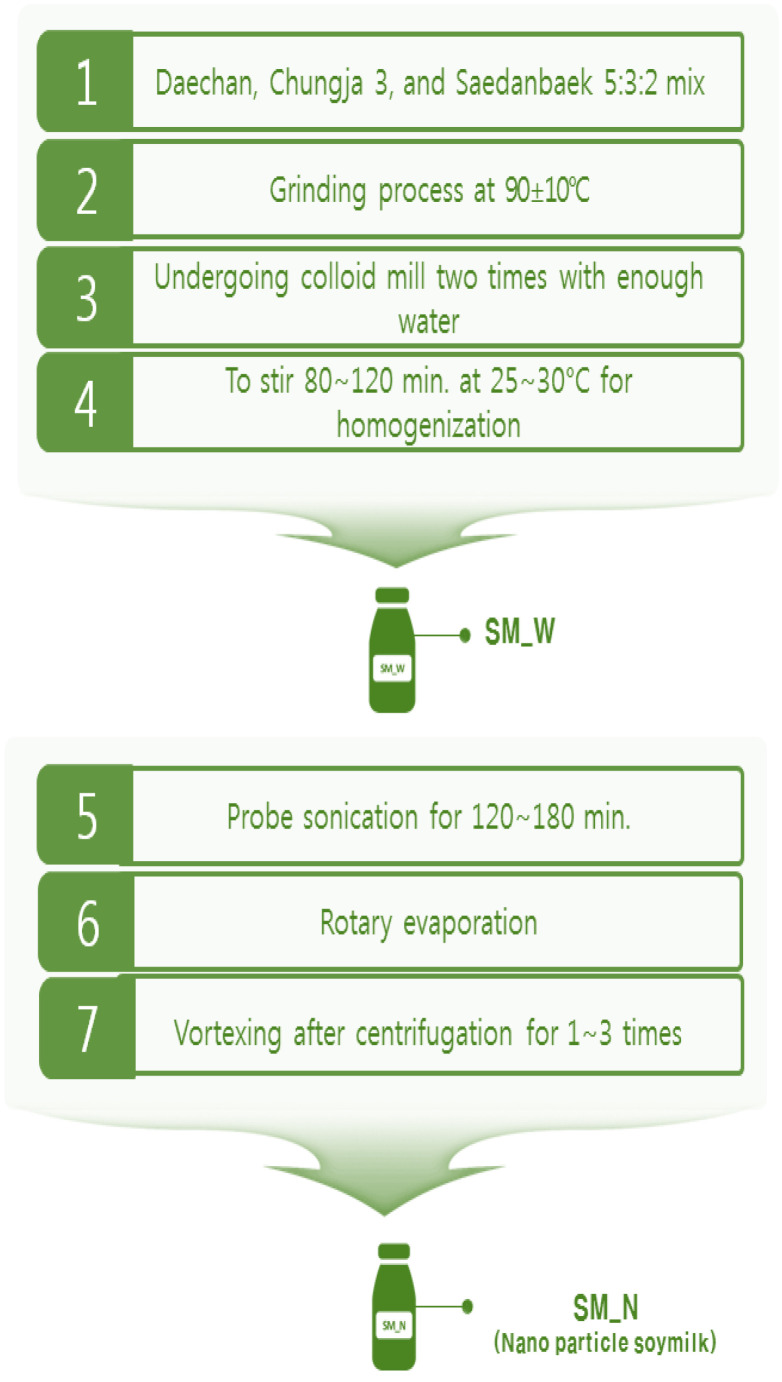
Laboratory soymilk production process.

The soy milks in the laboratory were developed from Daechan, Chungja 3, and Saedanbaek crops (20:12:8 = total 40 kg) by the Rural Development Administration. The soy milk processing technique is presented in Figure 1. It was produced by soaking the soy beans for 24 h, grinding at 80 °C, and exposure to colloid mills twice by adding water to obtain a crude liquid, which was stirred in a homogenizer (HF-93, SMT Co., Ltd., Tokyo, Japan) at 1000× *g* and 75~78 °C for 40 min to obtain the final soy milk. The nanoparticle-containing soy milk was obtained by stirring liquid for at least 100 min, followed by sonication and evaporation of water in the rotary evaporator for 100 min or more, followed by vortexing up to three times after centrifugation. The final nanoparticle-containing soy milk was produced after spray drying. The laboratory soy milk was stored in a sanitized plastic bag under refrigeration at 1.7 °C.

### 2.3. Sensory Evaluation

#### 2.3.1. Sample Preparation

Soy milks were stored at 4 °C in a refrigerator and used at the time of experiment. Samples of soy milk (20 mL) were poured into 50 mL white paper cups, and covered with lids and served at room temperature (20 ± 5 °C). Panelists were not allowed to eat or drink anything other than water 1 h prior to the descriptive test. Panelists were instructed to rinse their mouths with spring water before and between each sample. The samples were coded with 3-digit random numbers and presented using a Latin square design to minimize the carry-over effects [23].

The descriptive analysis was conducted in triplicate for 3 days, and each session evaluating the sensory attributes of the five soymilks lasted approximately 1 h [13,20]. A 9-point scale (ranging from ‘1 = not at all’ to ‘9 = extremely strong’) was used. Panelists discussed the ratings of attributes with other panelists. Panelists took a 15-min break between the sample tests (after the first 2 samples were tested) to prevent sensory adaptations. All reference standards were presented at room temperature. Panelists were allowed to retest and change their rating during the evaluation when a panelist failed to remember a reference for a specific attribute. Panelists rated the intensities of one attribute at a time for the entire sample set, rinsed their mouths with spring water between each sample, and moved on to the next attribute to minimize sample variation.

#### 2.3.2. Panel Selection and Training

Eight panelists selected from the students of Konkuk University (3 males and 5 females, age range 21~24) with previous experience in evaluating soy products were selected according to their interest, availability, and their ability to articulate. The basic screening tests, such as a basic taste test, flavor, and aroma recognition test, and an intensity ranking test were conducted during one week to understand the basics of sensory evaluation by panelists [24].

A quantitative descriptive analysis [25] was used to evaluate the sensory characteristics of the five soy milks. The descriptive analysis included training sessions and primary evaluation. Panelists were trained for a period of 2 weeks in 90-min sessions 3 times a week. Six sessions were devoted to tasting soymilks and group discussion to develop attributes and references. Initially, panelists were exposed to a variety of soymilks including laboratory-developed soymilks as well as commercial brands to obtain an understanding of the basics of sensory evaluation and the procedures. The preliminary sensory characteristics, such as the appearance, smell, taste, flavor, and mouthfeel, were generated after testing a variety of soy milks. In the next session, they were asked to evaluate sensory differences among samples, and then they generated the terms of attributes and selected references by group discussion.

During the third session, the panelists defined descriptive attributes compared with standard references and the final descriptive attributes were confirmed based on a consensus of the standard attributes. The panelists gave ratings based on a 16-point intensity rating scale (0 = none; 15 = extremely strong) in each attribute.

Furthermore, the preliminary intensity test was conducted to accurately rate the standard intensities of each attribute in individual booths. Then, three sessions of individual booth evaluations of panels were completed to collect data for the study. A supplementary training session was held to minimize the differences in intensity rates between the panelists. 

#### 2.3.3. Development of a Soy Milk Lexicon

The 20 sensory attributes were determined according to appearance, smell/aroma, taste/flavor, and mouthfeel/texture (Table 2). Four descriptors, such as grayness, whiteness, brownness, and roughness, were developed to define the appearance. The aroma/odor was described as sweet, goso (nuttiness), raw soy bean, cooked soy bean, wheat flavor, and roasted soy bean. Sweet and salty terms were used to define taste and milk, whereas raw, cooked, and roasted soybeans indicated flavor. Mouthfeel/texture was defined by cohesiveness, coating, astringent, swallow, and particle features. The definitions and references of the 20 descriptive attributes were also discussed. Table 2 lists the sensory attributes, definitions, and references of soy milks analyzed in this study. Furthermore, a preliminary test of sensory intensity was conducted to rate the relative intensity of the attributes. A few panelists received supplementary training to reduce the deviation of intensity rating.

#### 2.3.4. Consumer Acceptance Test

The consumers (*n* = 100, females: 54, males: 46, age: 20~26) were recruited from the students enrolled at Konkuk University. The consumer test also conducted the same test condition as the descriptive test. Samples of soy milk (20 mL) were poured into 50 mL white paper cups, and covered with lids and served at room temperature (20 ± 5 °C). The consumer test questionnaire included general characteristics about consumer panelists, overall acceptability, appearance acceptability, color acceptability, flavor acceptability, taste acceptability, mouthfeel acceptability, goso (nuttiness), sweet, repeated use, and recommendations to others. Consumers rated their liking using a nine-point hedonic scale (ranging from 1 (suggesting intense dislike) to 9 (indicating intense like)) [15]. The consumers rinsed their mouth with water between samples to avoid residual effects. The five samples coded with a 3-digit random number were randomly presented to the consumers using a Williams Latin square design [23].

### 2.4. Statistical Analysis

All statistical analyses were performed using SPSS (Statistical Package for Social Science, ver. 25.0, Chicago, IL, USA) and SAS (ver. 9.4, SAS Institute, Cary, NC, USA). To determine the significant differences in sensory attributes between the samples, a one-way analysis of variance (ANOVA) was performed. Duncan’s multiple range test was conducted as a post hoc comparison (a = 0.05). Principal component analysis (PCA) was conducted to identify sensory attributes and samples. In addition, partial least square regression (PLSR) analysis was conducted to correlate samples, descriptive attributes, and consumer acceptability.

## 3. Results and Discussion

### 3.1. Descriptive Analysis

Table 3 shows the mean intensity of 20 sensory attributes for 5 soy milk samples. The 20 sensory attributes except for the coating attribute varied significantly among the samples (*p* < 0.001). Based on sample appearance, the SM_Y commercial sample showed the maximum whiteness, whereas SM_D and SM_J showed the highest scores of brownness and grayness, respectively (*p* < 0.001). In terms of odor attributes, SM_D showed the highest score for sweetness, and SM_W scored the highest value for goso flavor (nuttiness) and cooked soy bean odor among the five types of soy milk (*p* < 0.001). Hwang and Hong [20] analyzed the sensory components of goso flavor (nuttiness) via descriptive analysis and consumer tests of 10 commercial types of soy milk. The results indicated that goso flavor (nuttiness) was positively correlated with cooked soy bean characteristics. A highly positive correlation existed between goso (nuttiness) and cooked soy bean attributes. With regard to taste and flavor attributes, the SM_J sample exhibited the lowest sweetness and the highest saltiness, and raw soybean flavor among five samples. In contrast, it yielded the lowest scores for cooked soybean and roasted soybean flavor among them (*p* < 0.001). SM_Y showed the lowest particle and the highest swallow of mouthfeel among the samples, which may be closely related to the highest degree of whiteness in the SM_Y sample. The SM_Y sample included very fine soybean solid particles compared with those of other types of soy milk. Several studies analyzed and investigated the descriptive attributes of soy milk. The descriptive sensory attributes of soymilk have been investigated by Torres-Penaranda and Reitmeier [13], Day N’Kouka et al. [3], and Rho et al. [25]. Torres-Penaranda and Reitmeier [13] presented 12 descriptive attributes based on commercial soymilk and soymilk processed from normal, lipoxygenase-free soybeans, and lipoxygenase-free soybeans stored for 15 months. The 12 descriptive attributes were ‘beany’ (raw as hexanal), ‘starch as flour’, and ‘sweet’ as dairy caramelized for aroma; ‘beany’, ‘grassy’, ‘sweet as green floral’, ‘painty’, ‘sweet as dairy caramelized’, ‘metallic’, and ‘bitter’ for flavor; and ‘astringent’ and ‘mouth coating’ for mouthfeel. Day N’Kouka et al. [3] developed 31 sensory terms of five commercial soymilks and 1 laboratory-prepared soymilk. The descriptive sensory terms included terms, such as cooked soy, green, nutty, roasted soy, caramel, malty, vanilla, and sweetness. Rho et al. [27] also reported 28 descriptive sensory attributes of 9 commercial soymilks. They developed the 28 descriptive attributes, which included ‘grayness’, ‘yellowness’, ‘clearness’, and ‘milky’ appearance; ‘opaque’, sweet’, ‘cooked chestnut’, ‘cooked soybean’, and ‘beany’ odor; ‘mild’, ‘salty’, ‘sour’, ‘sweet’, ‘bitter’, ‘greasy’, ‘savory’, ‘metallic’, ‘goso (nuttiness)’, ‘nutty’, ‘cooked chestnut’, ‘cooked soybean’, and ‘raw soybean’ flavor and taste; ‘coarse of particle’, ‘consistency’, ‘lubricity’, and ‘astringent’ mouthfeel; and ‘residual sensation’ and ‘coated’ after sensation. SM_N had high scores of cohesiveness and coating mouthfeel and lower swallowness mouthfeel than others. These results could be related with the encapsulation technique of the nanotechnique.

### 3.2. Principal Component Analysis of Soy Milk

**Figure 2:**
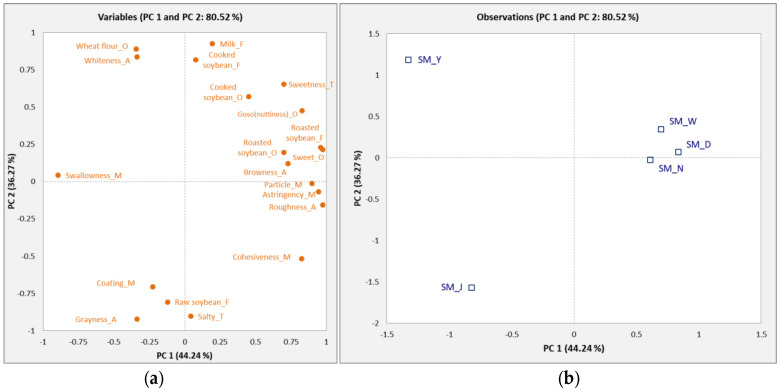
Principal component (PC) loadings and scores of the attributes (**a**) and five soymilk samples (**b**).

PCA was used to identify the relationship between the descriptive attributes and the samples in Figure 2. The PCA biplot represented 80.52% of the total variation with 44.24% (PC 1) and 36.27% (PC 2). The right side of PC 1 was associated with cooked soybean, sweet, roasted soybean, goso flavor (nuttiness), particle, astringent, and browny sensory attributes, and the SM_W, SM_N, and SM_D soymilk samples were closely related to those attributes. SM_W and SM_N showed similar sensory attributes because they were synthesized in the laboratory using the same ingredients. The negative side showed that raw soybean, coating, grayness, and salty attributes were related to the SM_J sample. Wheat flour and whiteness were located on the positive side of PC 2 and were closely associated with the SM_Y sample.

### 3.3. Consumer Acceptance

Table 4 presents the results of the consumer acceptability of the five soy milk samples. All soy milk samples were evaluated by 100 consumers based on the overall liking, appearance acceptability, color acceptability, flavor liking, taste acceptability, mouthfeel acceptability, goso flavor (nuttiness), sweet, repeated use, and recommendation. All attributes were not significantly different (*p* < 0.05). However, the SM_D sample generally showed the highest scores for the overall liking, flavor, taste, mouthfeel, sweet, repeated use, and recommendation. The synthetic SM_W displayed the highest score with regard to appearance among the samples. Otherwise, SM_N showed the highest color score based on nanotechnology.

Lee et al. [28] investigated the relationship between nutritional composition and soy milk palatability using nine soybean cultivars, including Daechan, Chungja 3, and Saedanbaek. The Daechan cultivar, which was an ingredient of the laboratory-made soy milk, had the highest overall palatability score. Our test samples of soy milk were developed without the addition of sweet components because we wanted to focus on the attributes of soy bean itself. Additionally, the other three commercial soy milks were also prepared as unsweetened ones. Therefore, the low average scores of the consumer tests may be closely related to the unsweetened soy milk samples. Several studies were used to analyze the soy milk regarding consumer interest [12,14,15,18,19]. A few such studies indicated that sweet-tasting soy milk topped consumer liking [12,18]. Vanilla flavor was also another preferred attribute among consumers reported in Villegas et al. [12] and Nti and Larweh [14]. Nti and Larweh [14] analyzed a consumer test of soy milk in Ghana and found that the addition of flavors, such as vanilla, banana, coffee, or chocolate, improved the consumer acceptability of soy milk. These studies found that consumers generally preferred a darker color, higher viscosity, sweet taste, and increased vanilla flavor.

### 3.4. Relationship between Descriptive Attributes and Consumer Acceptability of Soy Milk

**Figure 3:**
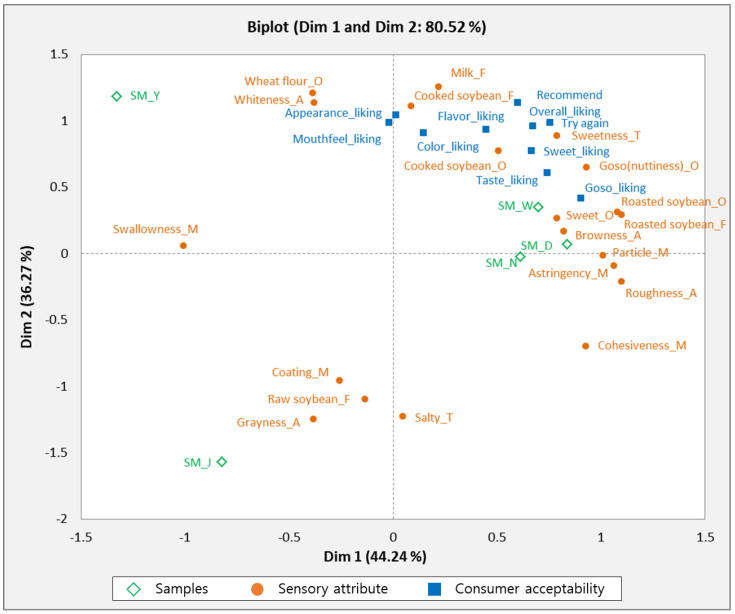
PLSR result from relationship between sensory attributes of five soymilks and consumer acceptability.

PLSR analysis was used to investigate the relationship between the descriptive attributes and consumer acceptability of the samples. Figure 3 shows the correlation between sensory attributes, consumer acceptability, and soy milk samples. The SM_W, SM_D, and SM_N samples were closely correlated with cooked soybean, goso flavor (nuttiness), sweet, browny texture, roasted soybean, particle, and astringent properties. These samples were also associated with the overall liking, sweet, color, flavor, taste, goso (nuttiness), repeated use, and positive recommendations. The results of this study showed that consumers preferred sweet, goso (nuttiness), roasted soybean, and cooked soybean attributes of soymilk, and sweetness was closely related with goso (nuttiness) and roasted soybean odor and flavor. The findings of this study are consistent with the study of Hwang and Hong [20], who also reported a positive relationship between goso (nuttiness) and sweet attributes of soy milk in consumer tests. SM_J was related to grayness, raw soybean, and coating attributes, which were located on the negative side of PLS 2. Based on consumer acceptability, SM_J scored the lowest in sensory attributes, suggesting that consumers dislike attributes of grayness, raw soybean flavor, and coating mouthfeel. Lawrence et al. [29] investigated the sensory attributes driving the interest in unflavored soymilks among different U.S. consumers, using descriptive analysis and consumer tests. They found that the least-liked sensory attributes were beany, green/grassy, and meaty/brothy flavors; bitter taste; and astringency. The SM_Y soy milk sample was associated with the odor of wheat flour and whiteness.

## 4. Conclusions

This study was conducted to identify the descriptive sensory characteristics and consumer acceptability of three commercial and two laboratory-developed soy milks, and analyzed the correlation between descriptive sensory characteristics and consumer acceptance of five soymilks. The descriptive analysis was conducted with eight trained panels. The 20 sensory attributes were determined according to appearance (grayness, whiteness, brownness, and roughness), smell (sweet, goso, raw soybean, cooked soybean, wheat flavor, and roasted soybean), taste (sweet, salty), flavor (milk, raw soybean, cooked soybean, and roasted soybean), and mouthfeel (cohesiveness, coating, astringent, swallow, and particles). Consumer acceptance was tested with 100 consumers and all attributes were not significantly different (*p* < 0.05). However, the SM_D sample generally showed the highest scores of overall liking, flavor, taste, mouthfeel, sweet, repeated use, and recommendation among all samples. Based on PLSR analysis, the SM_W, SM_D, and SM_N samples were closely related to cooked soybean, goso flavor (nuttiness), sweetness, browny texture, roasted soybean, particle, and astringent properties, and also correlated with overall liking, sweetness, color, flavor, taste, goso flavor (nuttiness), repeated use, and recommendation to others.

Overall, this study did not clearly show that the nano soy bean milk has improved sensory qualities compared to commercial ones. However, it presents the potential for the application of nanotechnology in soy milk development. The application of nanotechnology in the food industry remains a challenge because future studies provide guidance and rules regarding the public health benefits and risks for food nanotechnology. These results can be used as basic data for the application of the functionality of food nanotechnology and to expand the consumer market of soy milk.

## Figures and Tables

**Table 1:** Sample information.

Product Abbreviation	Ingredient	Random Sampling Numbers
SM_W	Daechan soybean:Cheongja 3 soybean:Saedanbaek soybean = 5:3:2 (20 kg:12 kg:8 kg)	185
SM_Y	Organic soymilk liquid (soybean solid, organic soybean, Australian), organic blackbean extract (solid, organic black bean, Korean)	257
SM_D	Soymilk liquid (soybean solid, soybean, Korean), small soybean powder (Korean), seaweed powder (United Kindom), sun-dried salt (Korean)	348
SM_N	Daechan soybean:Cheongja 3 soybean:Saedanbaek soybean = 5:3:2 (20 kg:12 kg:8 kg)	415
SM_J	Soybean liquid (soybean solid-imported), salt (Korean)	536

**Table 2:** Definition and standard reference of descriptive attributes of soymilk.

Attributes	Definition	Standard Reference
Appearance		
Grayness	Intensity of Grayness color	Strong: jongienara 120 colors 115/N5 (Jongienara, Seoul, Korea) (15) ^(1)^
Whiteness	Intensity of Whiteness color	Strong: jongienara 120 colors 143Y/NP (Jongienara, Seoul, Korea) (15)
Brownness	Intensity of Brownness color	Strong: jongienara 120 colors 1172YR/Gr (Jongienara, Seoul, Korea) (15)
Roughness	Intensity of Roughness	Strong: Powder of mixed grains 20 g with 100 mL water (15)
Odor (smell)		
Sweet	The smell associated with chocolate milk	Strong: Mixture 50 mL of chocolate milk with 50 mL water (15)
Goso (nuttiness)	The smell associated with Buckwheat tea	Strong: Buckwheat tea (15)
Cooked soybean	The smell associated with cooked soybean	Strong: 30 g of soybean that had been soaked for 3 h, cooked for 1 h, and then ground with 100 mL water (15)
Wheat flour	The smell associated with dough	Strong: Mix the flour 40 g and water 12.5 mL water to make dough (15)
Roasted soybean	The smell associated with roasted soybean	Strong: Bean flour (15)
Taste/Flavor		
Sweetness	Fundamental taste sensation elicited by sugars	Normal: 1.25% sucrose solution in spring water (8) Strong: 2.5% sucrose solution in spring water (15)
Salty	Fundamental taste sensation elicited by salts	Normal: 0.15% NaCl solution in spring water (8) ^(2)^ Strong: 0.3% NaCl solution in spring water (15)
Milk	Fundamental flavor sensation elicited by milks	Strong: Seoul milk (15)
Cooked soybean	Fundamental flavor sensation elicited by cooked soybean	Strong: 30 g of soybean that had been soaked for 3 h, cooked for 1 h, and then ground with 100 mL water (15)
Raw soybean	Fundamental flavor sensation elicited by raw soybean	Strong: Mixture of ground soybean (30 g soaked for 3 h and ground with 100 mL water) (15)
Roasted soybean	Fundamental flavor sensation elicited by roasted soybean	Strong: Bean flour (15)
Mouthfeel/Texture		
Cohesiveness	Degree to which liquid is viscous or thick	Normal: chocolate milk (Gana Milk) (8)
Coating	Degree to film coating the tongue	Normal: Seoul milk (8)
Astringency	Dryness perceived in the mouth	Strong: Mixture 15 g of green tea with 1 L water (15)
Swallowing	Degree to which water swallow in mouth	Strong: Soybean oil (15)
Particles	Degree to which particles of liquid	Normal: Powder of mixed grains (8)

^(1)^ 15 is the strongest intensity scores of standard reference; ^(2)^ 8 is the normal intensity scores of standard reference.

**Table 3:** Intensities of the descriptive attributes for five soymilks.

	SM_W	SM_Y	SM_D	SM_N	SM_J	*p*-Value ^(1)^		
						Sample	Panel	S × P ^(2)^
Appearance								
Whiteness_A	7.67 ± 2.25 ^b,(3)^	12.70 ± 1.54 ^a^	1.85 ± 1.32 ^c^	7.26 ± 2.19 ^b^	1.11 ± 0.58 ^c^	<0.001	<0.001	0.866
Browness_A	4.85 ± 2.41 ^b^	1.85 ± 1.17 ^c^	14.37 ± 0.88 ^a^	5.59 ± 2.68 ^b^	1.37 ± 1.21 ^c^	<0.001	0.104	0.980
Grayness_A	3.48 ± 1.65 ^b^	1.33 ± 0.48 ^c^	1.67 ± 2.32 ^c^	4.11 ± 2.08 ^a^	14.59 ± 0.98 ^a^	<0.001	0.053	0.906
Roughness_A	12.93 ± 2.38 ^ab^	1.48 ± 1.55 ^d^	12.22 ± 2.49 ^b^	13.52 ± 1.83 ^a^	6.33 ± 2.54 ^c^	<0.001	0.034	0.014
Odor (smell)								
Sweet_O	4.96 ± 2.86 ^b^	3.07 ± 1.52 ^c^	11.44 ± 1.74 ^a^	4.81 ± 2.59 ^b^	2.00 ± 1.96 ^c^	<0.001	0.347	0.216
Goso (nuttiness)_O	10.00 ± 2.73 ^a^	4.48 ± 2.17 ^d^	8.41 ± 2.68 ^b^	6.56 ± 3.12 ^c^	2.00 ± 1.94 ^e^	<0.001	0.808	0.981
Cooked soybean_O	13.04 ± 1.74 ^a^	7.89 ± 2.90 ^c^	5.48 ± 2.89 ^d^	10.52 ± 2.52 ^b^	3.19 ± 2.53 ^e^	<0.001	0.745	0.525
Wheat flour_O	7.63 ± 2.10 ^b^	10.96 ± 3.02 ^a^	4.96 ± 2.26 ^c^	7.15 ± 2.35 ^b^	3.78 ± 3.19 ^c^	<0.001	0.578	0.059
Roasted soybean_O	7.15 ± 2.03 ^b^	2.63 ± 1.60 ^c^	8.78 ± 3.20 ^a^	7.26 ± 1.79 ^b^	2.04 ± 1.32 ^c^	<0.001	0.481	0.267
Taste/Flavor								
Sweetness_T	7.70 ± 2.05 ^a^	5.37 ± 2.62 ^b^	7.56 ± 2.64 ^a^	5.56 ± 2.03 ^b^	2.41 ± 1.67 ^c^	<0.001	0.022	0.013
Salty_T	4.74 ± 1.53 ^c^	2.70 ± 2.52 ^d^	9.33 ± 2.47 ^b^	5.22 ± 2.17 ^c^	12.30 ± 1.94 ^a^	<0.001	<0.001	0.084
Milk_F	7.52 ± 2.06 ^b^	9.22 ± 2.72 ^a^	8.85 ± 2.43 ^a^	7.41 ± 2.04 ^b^	4.15 ± 2.49 ^c^	<0.001	0.490	0.441
Raw soybean_F	5.19 ± 1.96 ^b^	3.74 ± 3.18 ^c^	3.52 ± 1.45 ^c^	5.30 ± 2.15 ^b^	6.67 ± 1.62 ^a^	<0.001	0.011	0.055
Cooked soybean_F	10.56 ± 2.21 ^a^	10.74 ± 3.21 ^a^	5.93 ± 1.94 ^b^	10.19 ± 2.39 ^a^	4.11 ± 1.67 ^c^	<0.001	0.009	0.024
Roasted soybean_F	9.04 ± 2.58 ^a^	3.15 ± 1.70 ^b^	8.70 ± 2.52 ^a^	8.41 ± 2.55 ^a^	2.89 ± 1.45 ^b^	<0.001	0.911	0.330
Mouthfeel/Texture								
Cohesiveness_M	9.81 ± 2.34 ^ab^	6.59 ± 1.53 ^c^	9.89 ± 1.48 ^ab^	10.70 ± 2.52 ^a^	9.44 ± 1.25 ^b^	<0.001	0.210	0.195
Coating_M	9.33 ± 2.22	9.04 ± 1.26	8.78 ± 2.21	9.48 ± 2.14	9.78 ± 1.95	0.336	0.006	0.461
Swallowness_M	3.96 ± 1.34 ^d^	11.37 ± 1.52 ^a^	6.89 ± 1.65 ^c^	3.78 ± 1.05 ^d^	9.30 ± 2.16 ^b^	<0.001	0.002	0.347
Particle_M	10.74 ± 2.07 ^a^	1.59 ± 0.89 ^d^	7.26 ± 1.79 ^b^	11.44 ± 1.69 ^a^	3.81 ± 1.98 ^c^	<0.001	0.002	0.841
Astringency_M	9.78 ± 1.91 ^a^	2.19 ± 1.18 ^d^	7.81 ± 1.94 ^b^	9.93 ± 1.90 ^a^	4.56 ± 1.87 ^c^	<0.001	<0.001	0.529

^(1)^*p*-value by ANOVA, ^(2)^ The value of S × P means the *p*-value by two-way ANOVA by the interaction between Sample and Panel. ^(3)^ Mean ± Standard Deviation, ^a–e^ Means values within the same row with the same alphabet superscripts do not differ significantly (*p* < 0.05).

**Table 4:** Consumer acceptability of five soymilks.

	SM_W	SM_Y	SM_D	SM_N	SM_J	*p*-Value ^(1)^
Overall_liking	4.23 ± 2.19 ^(2)^	4.13 ± 2.31	4.34 ± 2.06	4.13 ± 1.83	2.91 ± 1.93	0.544
Appearance_liking	5.59 ± 1.74	5.50 ± 2.41	4.47 ± 1.83	5.27 ± 1.80	4.32 ± 2.32	0.306
Color_liking	5.72 ± 1.91	5.69 ± 2.42	4.52 ± 1.93	6.06 ± 6.06	4.29 ± 2.31	0.463
Flavor_liking	4.18 ± 2.22	4.72 ± 2.16	5.38 ± 2.22	4.51 ± 2.05	3.30 ± 1.77	0.086
Taste_liking	3.16 ± 1.84	3.05 ± 1.85	4.05 ± 2.14	3.21 ± 1.80	2.47 ± 1.90	0.584
Mouthfeel_liking	3.97 ± 1.85	4.42 ± 2.21	4.47 ± 1.89	3.79 ± 1.80	3.65 ± 2.01	0.089
Goso_liking	4.11 ± 2.16	3.72 ± 2.03	4.91 ± 1.82	4.20 ± 1.84	3.41 ± 2.13	0.121
Sweet_liking	2.95 ± 1.76	2.90 ± 1.87	3.39 ± 2.03	2.86 ± 1.74	2.45 ± 1.59	0.057
Try again	3.04 ± 2.03	2.81 ± 1.95	3.16 ± 2.04	2.97 ± 1.88	2.08 ± 1.41	0.235
Recommend	3.13 ± 1.98	3.06 ± 1.97	3.46 ± 2.04	3.05 ± 1.77	2.40 ± 1.68	0.166

^(1)^*p*-value by One-way ANOVA, ^(2)^ Mean ± Standard Deviation.
